# Supplementary material for: Modeling of Disintegration and Dissolution Behavior of Mefenamic Acid Formulation Using Numeric Solution of Noyes-Whitney Equation with Cellular Automata on Microtomographic and Algorithmically Generated Surfaces
Source: Pharmaceutics. 2018 Dec 3;10(4):259. doi: 10.3390/pharmaceutics10040259 (PMC6321502; doi:10.3390/pharmaceutics10040259)
Supplement: Supplementary file 1 [file pharmaceutics-10-00259-s001.pdf]

pharmaceutics

Supplementary Materials: Modeling of Disintegration and Dissolution Behavior of Mefenamic Acid Formulation Using Numeric Solution of Noyes-Whitney Equation with Cellular Automata on Microtomographic and Algorithmically Generated Surfaces

Reiji Yokoyama, Go Kimura, Christian M. Schlepütz, Jörg Huwyler and Maxim Puchkov

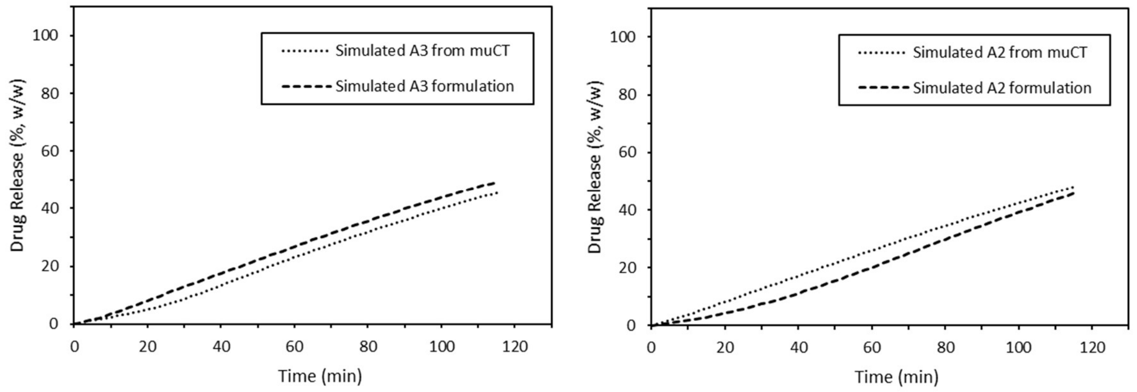

Figure S1: In silico drug release profiles obtained from simulations without the disintegration model of the algorithmically created tablet component arrangements, and the reconstructed matrices with the help of microtomography for formulations A3 (**left**) and A2 (**right**).
